# Expected Versus Experienced Health-Related Quality of Life Among Patients Recovering From Cancer Surgery: A Prospective Cohort Study

**DOI:** 10.1097/AS9.0000000000000060

**Published:** 2021-04-08

**Authors:** Nikhil Panda, Ian Solsky, Brandon J. Neal, Becky Hawrusik, Stuart Lipsitz, Carrie C. Lubitz, Chris Gibbons, Mary Brindle, Robert D. Sinyard, Jukka-Pekka Onnela, Christy E. Cauley, Alex B. Haynes

**Affiliations:** From the * Ariadne Labs, Brigham and Women’s Hospital, Harvard. T.H. School of Public Health, Boston, MA; † Department of Surgery, Massachusetts General Hospital, Boston, MA; ‡ Department of Surgery, Montefiore Medical Center, Albert Einstein College of Medicine, Bronx, NY; § Institute for Technology Assessment, Massachusetts General Hospital, Boston MA; ∥ Center for Integrative Systems from Patient-Reported Data in Cancer, MD Anderson, The University of Texas MD Anderson Cancer Center, Houston, TX; ¶ Department of Biostatistics, Harvard T.H. Chan School of Public Health, Boston, MA; # Department of Surgery and Perioperative Care, Dell Medical School, The University of Texas at Austin, Austin, TX.

**Keywords:** cancer surgery, expectations, health-related quality of life, patient-reported outcome measures

## Abstract

**Introduction:**

Patient expectations of the impact of surgery on postoperative health-related quality of life (HRQL) may reflect the effectiveness of patient-provider communication. We sought to compare expected versus experienced HRQL among patients undergoing cancer surgery.

**Methods:**

Adults undergoing cancer surgery were eligible for inclusion (2017–2019). Preoperatively, patients completed a smartphone-based survey assessing expectations for HRQL 1 week and 1, 3, and 6 months postoperatively based on the 8 short-form 36 (SF36) domains (physical functioning, physical role limitations, pain, general health, vitality, social functioning, emotional role limitations, and mental health). Experienced HRQL was then assessed through smartphone-based SF36 surveys 1, 3, and 6 months postoperatively. Correlations between 1- and 6-month trends in expected versus experienced HRQL were determined.

**Results:**

Among 101 consenting patients, 74 completed preoperative expectations and SF36 surveys (73%). The mean age was 54 years (SD 14), 49 (66%) were female, and the most common operations were for breast (34%) and abdominal (31%) tumors. Patients expected HRQL to worsen 1 week after surgery and improve toward minimal disability over 6 months. There was poor correlation (≤±0.4) between 1- and 6-month trends in expected versus experienced HRQL in all SF36 domains except for moderate correlation in physical functioning (0.50, 95% confidence interval [0.22–0.78], *P* < 0.001) and physical role limitations (0.41, 95% confidence interval [0.05–0.77], *P* = 0.024). Patients expected better HRQL than they experienced.

**Conclusions:**

Preoperative expectations of postoperative HRQL correlated poorly with lived experiences except in physical health domains. Surgeons should evaluate factors which inform expectations around physical and psychosocial health and use these data to enhance shared decision-making.

## Abstract 2

Mini-abstract: In a prospective cohort study, we used smartphone-based surveys to compare expected versus experienced postoperative health-related quality of life (HRQL) among patients undergoing cancer surgery. There was poor correlation in trends of expected versus experienced HRQL in all health domains except physical health constructs, where there was moderate correlation.

Supplemental Digital Content is available in the text.

## INTRODUCTION

Patient expectations for surgical recovery are informed by perceptions of the impact of surgery on health outcomes that matter to them.^1^ Both recovery expectations and lived experiences are increasingly being incorporated into measures of surgical quality and value, placing a greater emphasis on patient-centered care. This is true among patients undergoing treatment for cancer, where traditional surgical and oncologic outcomes are supplemented with measures of satisfaction and overall health-related quality of life (HRQL).^2^

Patient expectations for postoperative HRQL may reflect the quality of shared decision-making between patients and surgeons.^3^ Providing patients with high-quality information about surgery supports decision-making, allows patients to contextualize recovery, and informs expectations for postoperative HRQL. Despite the adoption of decision support interventions to enhance shared decision-making, patients and surgeons often have misaligned expectations.^4^ In cancer care, for example, clinicians often underestimate disability and overestimate HRQL among patients throughout treatment.^5^

The purpose of this study was twofold. First was to determine preoperative expectations of postoperative HRQL among patients undergoing cancer surgery; and the second was to measure and compare experienced HRQL throughout postoperative recovery with patients’ preoperative expectations. We hypothesized that patient expectations of postoperative HRQL would be poorly aligned with experiences during recovery. If so, these data would underscore the importance of measuring patients’ recovery expectations and supporting shared decision-making to more effectively align these expectations with lived experiences following cancer surgery.

## METHODS

### Patient Recruitment

The study protocol was approved by the Human Research Committee Institutional Review Board. Eligible patients included adult (≥18 years) patients scheduled to undergo operations for breast, skin/soft-tissue (eg, wide local excision of melanoma and lymphadenectomy), head and neck (eg, thyroidectomy and parathyroidectomy), or abdominal tumors from a single academic cancer center (July 2017–July 2019). Patients were approached for enrollment following consultation with their surgeon, approximately 1 week before surgery. Consenting patients were instructed to download the Beiwe smartphone application, the user-facing component of a platform designed and previously used by our team for smartphone-based digital phenotyping research in surgical patients.^6–8^ Given that all study interventions to assess HRQL were in English and delivered through this smartphone application, patients were excluded if they did not own a smartphone or were not fluent English-speakers.

At the time of enrollment, patients’ electronic health records were reviewed for the following demographics and disease characteristics: age, self-reported gender, self-reported race and ethnicity, body mass index (BMI, kg/m^2^), and cancer/treatment information (eg, neoadjuvant chemotherapy or radiation within 6 months of surgery, surgical pathology, and adjuvant treatment). During the 6-month study period, the electronic health records were periodically reviewed for perioperative details (eg, operative time, blood loss, postoperative morbidity, discharge disposition, and follow-up visits). All data were securely stored in Research Electronic Data Capture (REDCap).^9^

### Measurement of Expected and Experienced HRQL

At the time of enrollment, patients were electronically sent a survey via the Beiwe smartphone application to assess expectations for postoperative HRQL during recovery. Given the lack of psychometrically validated instruments to assess patient expectations longitudinally during perioperative care,^1^ this survey was developed by our research team comprised individuals with content expertise in surgical oncology and health services research. Additional review was provided by experts with extensive experience in qualitative research, HRQL survey methodology, and psychometrics. The design of the survey was informed by the Short Form Health Surveys (SF8 and SF36 version 1) and asked patients to quantify their expected health at 1 week and 1, 3, and 6 months after surgery.^10–12^ For each of these recovery times, patients were asked to estimate their future health in each of the 8 SF36 health domains: (1) physical functioning; (2) physical role limitations; (3) bodily pain; (4) general health; (5) vitality; (6) social functioning; (7) mental health; and (8) emotional role limitations. These domains capture patients’ expectations for their postoperative generic HRQL. To minimize survey burden and maximize feasibility, additional disease- or treatment-specific surveys measuring oncologic expectations after surgery were not distributed. A copy of the preoperative expectations survey is provided in Supplemental Digital Content 1, http://links.lww.com/AOSO/A26.

In addition to the preoperative expectations survey, patients were also electronically sent the SF36 survey via the Beiwe application at enrollment and at 1, 3, and 6 months after surgery to assess experienced HRQL during recovery. The SF36 has been used to assess longitudinal HRQL, including among patients undergoing elective cancer operations, and is sensitive to changes in each of the 8 health domains following surgery.^13^ Patients were excluded if they did not complete the preoperative expectations or SF36 surveys.

The primary endpoint in this study was to measure differences in trends of expected versus experienced HRQL among patients undergoing operations for cancer as measured by surveys delivered remotely through a smartphone platform.

### Data Management and Statistical Analysis

All survey responses were encrypted and stored in compliance with the Health Insurance Portability and Accountability Act. Baseline patient demographics and clinical outcomes data were summarized using means and SDs for continuous measures, or frequencies for categorical data. To measure differences in characteristics among survey responders and nonresponders, bivariate analyses using two-sample Student *t*-tests, Wilcoxon tests, Chi-squared tests, and Fisher exact tests were performed. Completion rates for the preoperative expectations and postoperative SF36 surveys were determined using the American Association for Public Opinion Research Standard Definitions Report.^14^ Only surveys initiated within 1 week of the assigned date and those with ≥75% completion were included in the final analyses.

To compare trends in expected versus experienced HRQL, correlation of 1- to 6-month observed differences in responses to the preoperative expectations and postoperative SF36 surveys were performed. Correlations were summarized using Spearman’s correlation coefficients for nonnormally distributed data, where correlations ≤±0.4 were considered poor, ±0.4 to 0.7 considered moderate, and ≥±0.8 considered strong.^15,16^ To assess for bias due to missing data (eg, differences in characteristics or postoperative outcomes among responders vs nonresponders to the postoperative SF36 surveys), an additional sensitivity analysis was performed. Specifically, a repeated-measure linear mixed model applying multiple imputation was fit to determine if there were differences in predicted versus observed HRQL. All analyses were performed using STATA (Version 15.1, StataCorp, College Station, TX) and SAS software (Version 9.4, SAS Institute, Cary, NC).

## RESULTS

### Study Population

**FIGURE 1.**
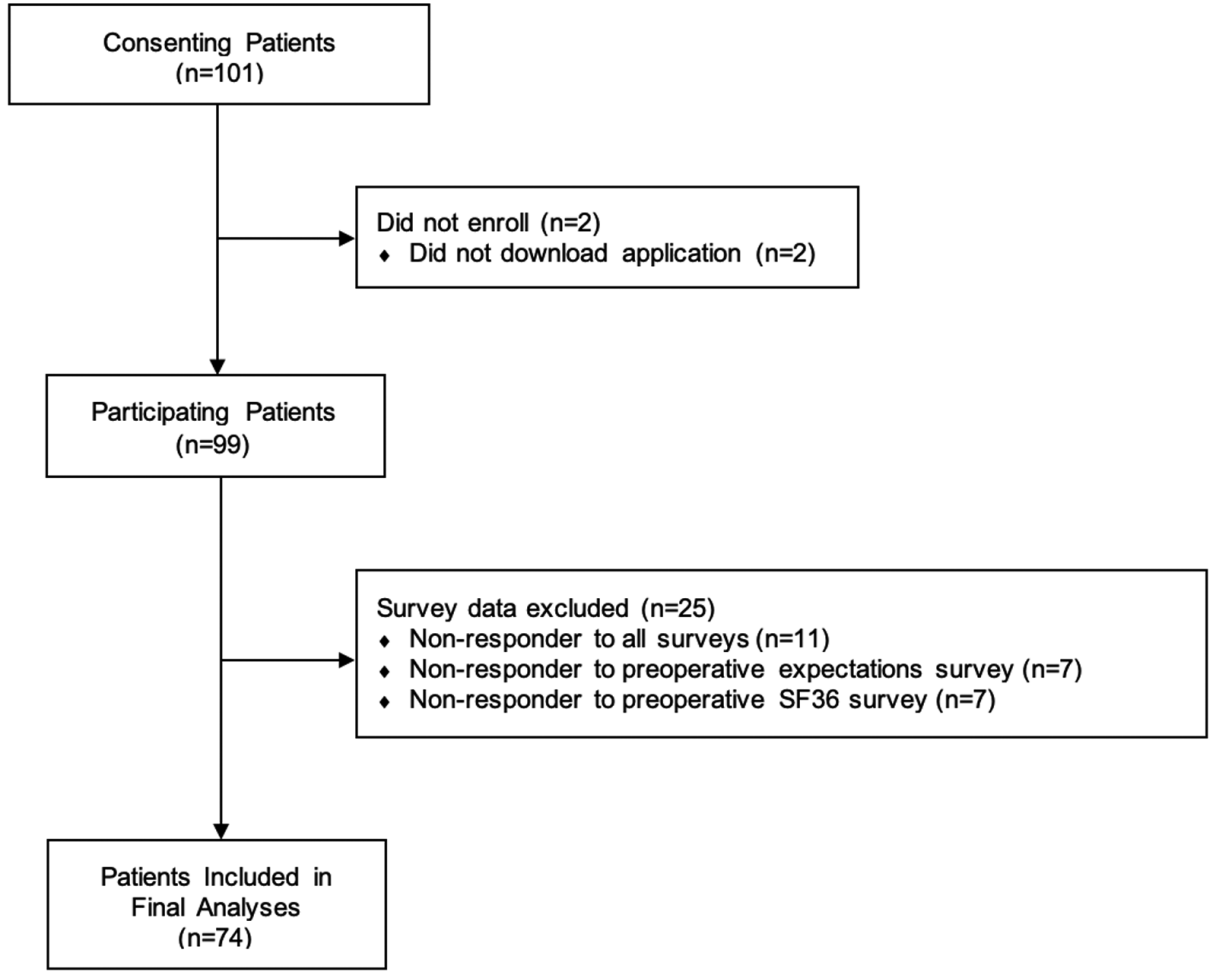
Flow diagram showing recruitment and reasons for data exclusion among participating patients. Only surveys completed within one week of the scheduled date and with ≥75% completion were included.

Among 101 patients who consented to participate, 74 completed both the preoperative expectations and SF36 surveys (73% completion rate, Fig. 1). Among these patients, the mean age was 54 years (SD 14), 49 (66%) were female, and the most common operations were performed for breast (34%) and abdominal (31%) tumors. Additional demographics, disease factors, operative details, postoperative outcomes, and adjuvant treatment details are shown in Table 1.

**TABLE 1.** Characteristics of Included Patients

	N = 74
Demographics and disease factors	
Age (y, mean, SD)	53.9 (13.9)
Female (n, %)	49 (66.2)
Race and ethnicity (n, %)	
Non-Hispanic White	64 (86.5)
Non-Hispanic Black	2 (2.7)
Hispanic	2 (2.7)
Asian	1 (1.3)
Other	2 (2.7)
Unknown	3 (4.1)
BMI (kg/m^2^, mean, SD)	27.8 (5.8)
Smartphone operating system (n, %)	
iPhone	61 (82.4)
Android	13 (17.6)
Tumor primary site (n, %)	
Breast	25 (33.8)
Head and neck	11 (14.9)
Abdominal	23 (31.1)
Skin/soft tissue	15 (20.3)
Neoadjuvant treatment (n, %)	
Chemotherapy	16 (21.6)
Radiation	8 (10.8)
Operative details and postoperative outcomes	
ASA classification* (mean, SD)	2.1 (0.5)
Operative time (min, mean, SD)	160.9 (133.1)
Blood loss* (mL, mean, SD)	120.2 (236.6)
Length of stay (d, mean, SD)	2.7 (3.1)
Discharge with home services (n, %)	27 (36.5)
Postoperative morbidity (n, %)	12 (16.2)
Readmission (n, %)	9 (12.2)
Return to operating room (n, %)	14 (18.9)
Adjuvant treatment (n, %)	
Chemotherapy	12 (16.2)
Radiation	13 (17.6)
Immunotherapy	6 (8.1)
Number of follow-up visits with surgeon* (mean, SD)	2.2 (2.4)
Number of follow-up visits with any provider (mean, SD)	7.8 (6.0)

*ASA and number of follow-up visits recorded for 73/74 patients. Blood loss recorded for 62/74 patients.

ASA indicates American Society of Anesthesiology.

There were no differences in baseline characteristics and disease factors comparing the 74 patients who completed the preoperative expectations and SF36 surveys versus noncompleters (Table 2). During the postoperative study period, there were 42 (42%) patients who completed the SF36 at 1 month after surgery; 33 (33%) patients at 3 months after surgery; and 24 (24%) patients at 6 months after surgery. The demographics, disease factors, operative details, and postoperative outcomes of patients at each time during the postoperative study period are summarized in Table 3.

**TABLE 2.** Preoperative Survey Completers Versus Noncompleters

	Completers (n = 74)	Noncompleters (n = 27)	*P*
Demographics and disease factors			
Age (y, mean, SD)	53.9 (13.9)	48.9 (10.5)	0.092
Female (n, %)	49 (66.2)	18 (66.7)	0.966
Race and ethnicity (n, %)			
Non-Hispanic White	64 (86.5)	23 (85.2)	0.937
Non-Hispanic Black	2 (2.7)	1 (3.7)	
Hispanic	2 (2.7)	1 (3.7)	
Asian	1 (1.3)	1 (3.7)	
Other	2 (2.7)	0 (0.0)	
Unknown	3 (4.1)	1 (3.7)	
BMI (kg/m^2^, mean, SD)	27.8 (5.8)	26.2 (5.5)	0.186
Smartphone operating system (n, %)			
iPhone	61 (82.4)	22 (81.5)	0.771
Android	13 (17.6)	5 (18.5)	
Tumor primary site (n, %)			
Breast	25 (33.8)	9 (33.3)	0.930
Head and neck	11 (14.9)	3 (11.1)	
Abdominal	23 (31.1)	10 (37.0)	
Skin/soft tissue	15 (20.3)	5 (18.5)	
Neoadjuvant treatment (n, %)			
Chemotherapy	16 (21.6)	8 (29.6)	0.403
Radiation	8 (10.8)	4 (14.8)	0.729

Demographics, disease factors, operative details, and postoperative outcomes among completers versus noncompleters of preoperative surveys.

ASA indicates American Society of Anesthesiology; BMI, body mass index.

**TABLE 3.** Characteristics of Survey Completers During the Study Period

	Preoperative (n=74)	Postoperative		
		Month 1(n=42)	Month 3(n=33)	Month 6(n=24)
Demographics and disease factors				
Age (y, mean, SD)	53.9 (13.9)	53.6 (11.8)	58.0 (9.4)	59.7 (9.7)
Female (n, %)	49 (66.2)	30 (71.4)	23 (69.7)	16 (66.7)
Race and ethnicity (n, %)				
Non-Hispanic White	64 (86.5)	41 (97.6)	33 (100.0)	23 (95.8)
Non-Hispanic Black	2 (2.7)	0 (0.0)	0 (0.0)	0 (0.0)
Hispanic	2 (2.7)	0 (0.0)	0 (0.0)	0 (0.0)
Asian	1 (1.3)	0 (0.0)	0 (0.0)	0 (0.0)
Other	2 (2.7)	0 (0.0)	0 (0.0)	0 (0.0)
Unknown	3 (4.1)	1 (2.4)	0 (0.0)	1 (4.2)
BMI (kg/m^2^, mean, SD)	27.8 (5.8)	26.6 (5.6)	27.0 (5.5)	26.0 (5.9)
Smartphone operating system (n, %)				
iPhone	61 (82.4)	42 (100)	32 (97.0)	23 (95.8)
Android	13 (17.6)	0 (0.0)	1 (3.0)	1 (4.2)
Tumor primary site (n, %)				
Breast	25 (33.8)	18 (42.9)	13 (39.4)	9 (37.5)
Head and neck	11 (14.9)	6 (14.3)	5 (15.1)	6 (25.0)
Abdominal	23 (31.1)	12 (28.6)	9 (27.3)	6 (25.0)
Skin/soft tissue	15 (20.3)	6 (14.3)	6 (18.2)	3 (12.5)
Neoadjuvant treatment (n, %)				
Chemotherapy	16 (21.6)	9 (21.4)	6 (18.2)	6 (25.0)
Radiation	8 (10.8)	3 (7.1)	3 (9.1)	3 (12.5)
Operative details and postoperative outcomes				
ASA classification* (mean, SD)	2.1 (0.5)	2.0 (0.5)	2.1 (0.4)	2.1 (0.5)
Operative time (min, mean, SD)	160.9 (133.1)	157.3 (141.6)	160.1 (134.9)	177.7 (147.1)
Blood loss* (mL, mean, SD)	120.2 (236.6)	109.6 (248.9)	119.8 (276.8)	137.6 (307.6)
Length of stay (d, mean, SD)	2.7 (3.1)	3.0 (3.1)	3.2 (3.3)	3.2 (2.9)
Discharge with home services (n, %)	27 (36.5)	18 (42.9)	16 (48.5)	14 (58.3)
Postoperative morbidity (n, %)	12 (16.2)	6 (14.3)	5 (15.1)	6 (25.0)
Readmission (n, %)	9 (12.2)	4 (9.5)	2 (6.1)	3 (12.5)
Return to operating room (n, %)	14 (18.9)	9 (21.4)	7 (21.2)	5 (20.8)
Follow-up visits: surgeon* (mean, SD)	2.2 (2.4)	2.2 (2.8)	2.3 (2.9)	2.9 (3.2)
Follow-up visits: any provider (mean, SD)	7.8 (6.0)	9.1 (6.4)	8.0 (5.3)	8.7 (5.5)

*ASA and number of follow-up visits recorded for 73/74 participants preoperatively and 41/42 responders at postoperative month 1; blood loss for 62/74 participants preoperatively, 37/42 participants at postoperative month 1, 29/33 participants at postoperative month 3, and 21/24 participants at postoperative month 6.

ASA indicates American Society of Anesthesiology; BMI, body mass index.

### Expected and Experienced HRQL

**FIGURE 2.**
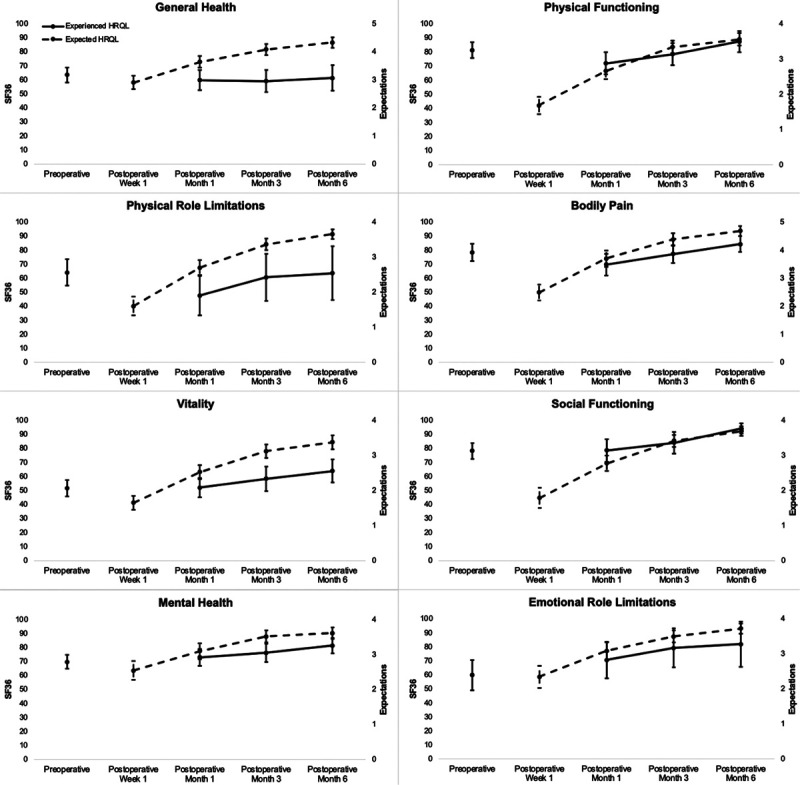
Trends in expected versus experienced postoperative HRQL. Observed results from the preoperative expectations (dashed line) and postoperative SF36 surveys (solid line) with 95% CIs provided at each postoperative time point (ie, 1, 3, and 6 months) for the 8 SF36 health domains.

Figure 2 shows the results of the expectations and SF36 surveys summarizing patients’ expected and experienced HRQL, respectively, in each of the 8 SF36 health domains. Across all health domains, patients seemed to expect that their HRQL would worsen compared with their preoperative baseline at 1 week postoperatively and then improve steadily during recovery toward minimal disability by the end of the 6-month study period. Comparing preoperative expected versus experienced HRQL as measured by the results of the SF36 at months 1, 3, and 6 postoperatively, there was poor correlation of 1- to 6-month trends in 6 of the 8 health domains (eg, general health domain: correlation coefficient −0.15, 95% confidence interval [CI] [−0.48 to 0.18], *P* = 0.369, Table 4). There was moderate correlation in trends of expected and experienced HRQL only in the physical functioning (correlation coefficient 0.50, 95% CI [0.22–0.78], *P* < 0.001) and physical role limitations domains (correlation coefficient 0.41, 95% CI [0.05–0.77], *P* = 0.024). The results of the sensitivity analysis to assess for bias due to missing data (eg, nonresponders to the postoperative SF36 surveys) are shown in Table 5. There were no significant differences in observed versus predicted HRQL at 1, 3, and 6 months postoperatively (eg, 6-month observed vs predicted general health domain scores: 61.3, 95% CI [52.2–70.3] vs 61.4, 95% CI [55.6–67.3]).

**TABLE 4.** Correlation of Trends in Expected Versus Experienced HRQL Spearman correlation coefficients for 1- to 6-month differences in expected versus experienced HRQL by health domain.

Health Domain	Correlation Coefficient [95% CI]	*P*
General health	−0.15 [−0.48 to 0.18]	0.369
Bodily pain	0.27 [−0.04 to 0.57]	0.089
Physical functioning	0.50 [0.22 to 0.78]	<0.001
Emotional role limitations	0.17 [−0.21 to 0.55]	0.367
Physical role limitations	0.41 [0.05 to 0.77]	0.024
Social functioning	0.19 [−0.11 to 0.50]	0.216
Vitality	0.21 [−0.16 to 0.57]	0.277
Mental health	0.35 [−0.01 to 0.70]	0.055

Cell values reflect observed and predicted (ie, results from linear mixed model) mean domain scores and 95% CIs.

**TABLE 5.** Observed and predicted HRQL as measured by the SF36 preoperatively and at postoperative months 1, 3, and 6

Health Domain	Preoperative		Postoperative Month 1		Postoperative Month 3		Postoperative Month 6	
	Observed	Predicted	Observed	Predicted	Observed	Predicted	Observed	Predicted
Vitality	51.6 [45.9–57.4]	51.6 [45.9–57.3]	52.0 [45.2–58.9]	50.8 [44.4–57.3]	58.3 [49.6–67.0]	54.0 [46.4–61.6]	64.0 [55.8–72.1]	58.8 [52.5–65.0]
Social functioning	78.2 [72.6–83.8]	78.2 [72.6–83.8]	78.6 [70.6–86.5]	78.5 [70.9–86.2]	84.1 [76.3–91.9]	81.2 [73.6–88.7]	94.3 [90.8–97.7]	94.1 [90.9–97.3]
Emotional role limitations	59.8 [48.9–70.7]	59.3 [48.4–70.2]	70.6 [57.6–83.7]	65.5 [53.4–77.5]	79.3 [65.4–93.2]	68.6 [56.3–81.0]	81.9 [65.9–98.0]	79.1 [66.0–92.2]
Physical role limitations	64.2 [54.6–73.7]	64.2 [54.7–73.7]	47.6 [33.5–61.8]	47.0 [33.6–60.4]	60.6 [43.9–77.3]	60.7 [45.3–76.1]	63.5 [44.4–82.7]	67.0 [51.6–82.4]
Bodily pain	78.4 [72.2–84.7]	78.4 [72.3–84.6]	69.7 [61.9–77.5]	69.2 [61.6–76.7]	77.2 [71.0–83.5]	76.3 [70.7–82.0]	84.4 [78.9–89.9]	84.5 [79.8–89.2]
Mental health	69.8 [64.8–74.7]	69.8 [64.9–74.7]	73.0 [67.0–78.9]	75.9 [70.7–81.1]	76.1 [69.7–82.5]	75.8 [70.3–81.2]	81.3 [75.9–86.8]	80.8 [76.7–84.9]
Physical functioning	81.4 [75.8–86.9]	81.4 [75.8–86.9]	71.9 [64.1–79.7]	68.2 [60.5–75.9]	78.5 [70.7–86.3]	76.8 [69.9–83.6]	87.5 [79.9–95.0]	84.9 [79.5–90.3]
General health	63.5 [58.2–68.8]	63.5 [58.2–68.8]	59.8 [52.7–66.9]	59.6 [53.2–66.0]	59.2 [51.4–66.9]	62.1 [55.8–68.3]	61.3 [52.2–70.3]	61.4 [55.6–67.3]

## DISCUSSION

In this study, we used smartphone-based surveys to measure and compare the expected and experienced postoperative HRQL among 74 patients undergoing cancer surgery. Across 8 health domains, patients expected HRQL to worsen 1 week after surgery and improve toward minimal disability at 6 months postoperatively. There was poor correlation in trends of expected versus experienced HRQL in all health domains except in constructs related to physical health (eg, physical functioning and physical role limitations), where there was moderate correlation. Patients generally expected better HRQL than what they experienced postoperatively.

### Patient Expected Versus Experienced HRQL

Previous research evaluating patient expectations for surgical recovery has focused on associations with postoperative patient satisfaction, disease-specific outcomes, and longitudinal HRQL.^17^ A common finding is that HRQL generally worsens immediately following surgery, most notably in constructs related to physical health, and progressively improves toward baseline after several months of recovery.^13^ This biphasic pattern in recovery is aligned with what the patients in the current study reported. Few investigations, however, have measured the concordance between recovery expectations and lived experiences following surgery, especially using patient-centered outcome measures.^18^ In one of the largest prospective studies of 454 patients undergoing orthopedic, vascular, and thoracic cancer operations, Mangione et al^13^ collected HRQL outcomes using the SF36 up to 1 year following surgery and found that the observed trends agreed with expectations for recovery, where expectations were defined by the clinical investigators. Importantly, the results of the current study extend these findings by comparing postoperative HRQL with preoperative expectations for recovery, where expectations were specified by patients.

We observed poor correlation between trends in expected versus experienced HRQL in nearly all health domains, where patients generally expected better HRQL than what they experienced. There are several potential explanations for these findings. Each warrants discussion as misalignment between expectations and experiences place patients at risk for worse satisfaction with treatment, poorer relationships with providers and health systems, and adverse health outcomes.^17^

First, patients’ recovery expectations represent perceptions of the impact of surgery on postoperative HRQL as informed, in part, by surgeons. Therefore, discordance between expectations and lived experiences may reflect the quality of shared decision-making. Elwyn et al^3^ previously framed effective shared decision-making as empowering patients through exchanging information regarding treatment outcomes and supporting their process of decision-making. In doing so, patients’ decisions are anchored in realistic expectations for postoperative HRQL. This may explain our observation of moderate correlation in trends of expected and experienced HRQL in constructs related to physical health. Given that many postoperative metrics are perceived as surrogates of physical health or functional independence (eg, physical activity and return to work), surgeons may prioritize or more effectively set expectations regarding the impact of surgery in physical rather than emotional or psychosocial health domains.

Second, it is possible that surgeons communicate more effectively about pathophysiology and operative treatments than the effects of surgery on postoperative HRQL. A substantial body of the prior literature has demonstrated that while surgeons are active in counseling patients, we rarely empathize with patients and infrequently explore or advise them on the potential psychosocial impact of surgical treatment options.^19,20^ These represent areas for ongoing improvement in surgeon–patient communication, which may lead to more appropriate framing of expectations for recovery. Last, surgeons may lack the data or tools necessary to fully understand the nuanced impact of surgery on HRQL, especially in terms of postoperative physical or psychosocial health. As a result, patients may enter treatment with unrealistic or unknown expectations for recovery. This is supported by previous studies among patients with breast and prostate cancer, where treating physicians substantially underestimated disease-related disability and overestimated HRQL when compared with their patients’ self-report, a finding that has also been demonstrated outside of oncologic patient populations.^4,5,18,21–23^

The lack of nuanced data describing the impact of surgery on postoperative psychosocial HRQL underscores the ongoing unmet need to develop and collect longitudinal, patient-centered recovery metrics following cancer surgery. The current implementation of patient-reported outcome measures has prioritized the patient perspective throughout preoperative and postoperative care.^2^ In addition, our research team has previously evaluated the role of patient generated health data captured from mobile health technologies, such as smartphone sensors (eg, accelerometer and global positioning system), to develop novel recovery metrics. These and other similar tools allow patients and surgeons to capture not only physical, but potentially also mental, emotional, social, and behavioral health throughout episodes of surgical care. Combining these data with traditional surgical and oncologic outcome measures at the time of consultation will ultimately allow surgeons and patients to appropriately set expectations for recovery.

### Measuring Expectations

An important consideration when contextualizing the lack of agreement between trends in expected versus experienced HRQL relates to the method of measuring patient expectations. While postoperative HRQL was assessed using the SF36—an instrument that has been extensively psychometrically evaluated—preoperative expectations of postoperative HRQL were measured using a survey designed for the purposes of this study. This was due to the lack of an available, psychometrically tested instrument during the study period. In a systematic review of 60 studies on patient expectations for surgical recovery, Waljee et al^17^ found that 83% of studies used nonvalidated methods (eg, ad hoc questionnaires, modified surveys, or qualitative methods) to measure expectations when lacking disease- or operation-specific instruments. The first report of a psychometrically tested general measure of patient expectations was recently introduced by Barth et al^1^ in 2019. Recognizing the associations of unmet patient expectations of postoperative satisfaction, patient–provider relationships, and health outcomes, there is a significant need and opportunity for surgeons to develop and implement methods to measure patient expectations. In doing so, surgeons can better understand patient preferences that contribute to perceptions of postoperative HRQL outcomes, and use these data to inform tools to enhance shared decision-making around the time of surgery.^24^

### Limitations

The findings of this study must be interpreted in the context of the following important limitations. First, this was a relatively small and heterogenous patient population including those undergoing surgery for breast, skin/soft tissue, head and neck, and abdominal tumors. As such, we were unable to perform additional sensitivity analysis to explore the lack of concordance between expected and experienced HRQL (eg, by type of cancer surgery, postoperative complication, length of stay, or adjuvant treatment). The study population demography was also relatively ethnically homogenous (86.5% non-Hispanic white patients), which may limit the generalizability of our findings. Future work must strive to include more diverse patient populations. The wide ownership and usage of smartphones across social determinants of health underscores the possibility of performing studies with similar methods in a more generalizable patient population. A second limitation is that preoperative and postoperative HRQL were assessed using the SF36 rather than disease-, treatment-, and cancer-specific HRQL instruments. We chose a generic health instrument as it applied to a heterogenous sample of patients regardless of differences in individual treatment plans. This minimized the burden associated with completing multiple surveys. Third, despite the relatively high completion rate of the preoperative surveys, there was loss to follow up when assessing experienced HRQL at 1, 3, and 6 months postoperatively. However, there were no significant differences in the characteristics of survey completers versus noncompleters at baseline, and the results of the sensitivity analysis comparing observed versus predicted postoperative HRQL demonstrated similar findings. While this suggests that there was minimal bias due to non-response to the postoperative SF36, there may be additional, unmeasured sources of bias in these trends of postoperative HRQL. Fourth, while we contextualized postoperative HRQL in terms of patients’ expectations, we did not assess surgeon expectations, which would allow us to comment on the effectiveness of shared decision-making. Subsequent studies will incorporate not only patient expectations for HRQL, but also the perspectives of surgeons and caregivers.

Last, there are several limitations of the survey to measure preoperative expectations of postoperative HRQL, which was designed for this study. The survey was not previously validated, although it did appear to have face validity when reviewed by experts in surgical oncology, psychometrics, and survey methodology. Furthermore, the survey captured expectations in terms of general HRQL, but did not collect information on oncologic expectations (eg, being cancer free). Patients may have answered the postoperative SF36 differently based on the oncologic outcome of their treatment. While this study’s results are significant for finding that preoperative expectations of postoperative HRQL correlated poorly with lived experiences, future studies can consider the underlying reasons for this finding, which may include misalignment of oncologic expectations.

## CONCLUSION

Patients experienced HRQL outcomes following cancer surgery which were poorly correlated with preoperative expectations. Recognizing that patient expectations reflect, in part, the information provided by surgical care teams, the results of this study underscore the need to strive for greater concordance between expected and experienced postoperative HRQL. Failing to do so may result in poorer patient satisfaction, patient–provider relationships, and overall health outcomes. There is an opportunity for surgeons to contribute to the development and implementation of tools to capture expected HRQL outcomes among patients, and use these data to strengthen shared decision-making prior to surgery.

## ACKNOWLEDGMENTS

N.P. and I.S. contributed equally as first authors. C.E.C. and A.B.H. contributed equally as senior authors.

N.P., I.S., and A.H. participated in research design, performance of the research, data analysis, contributed to new analytic tools, writing of the paper, critical review of the paper, and approval for submission. B.N. participated in data analysis, contributed to new analytic tools, critical review of the paper, and approval for submission. B.H., S.L., C.L., and C.C. participated in data analysis, contributed to new analytic tools, writing of the paper, critical review of the paper, and approval for submission. C.G., M.B., R.S. participated in data analysis, critical review of the paper, and approval for submission. J.-P.O. participated in research design, performance of the research, critical review of the paper, and approval for submission.

## Supplementary Material
