# Supplementary material for: Expected Versus Experienced Health-Related Quality of Life Among Patients Recovering From Cancer Surgery: A Prospective Cohort Study
Source: Ann Surg Open. 2021 Apr 8;2(2):e060. doi: 10.1097/AS9.0000000000000060 (PMC8221715; doi:10.1097/AS9.0000000000000060)
Supplement: Supplementary file 1 [file as9-2-e060-s001.pdf]

Supplemental Digital Content, Materials and Methods – Expectations Survey. Below is the survey designed for this study to assess expectations for postoperative health-related quality of life among patients scheduled to undergo cancer operations.

The following eight questions will ask you about different aspects of your health and how you anticipate you will feel 1 week, 1 month, 3 months, and 6 months after surgery. Please choose one item for each time period that best describes how you think you will feel at that time.

1. I anticipate my overall health will be [Insert time frame: 1 week, 1 month, 3 months, 6 months after surgery]:
    a. Excellent
    b. Very good
    c. Good
    d. Fair
    e. Poor
    f. Very Poor [Domain: General health perceptions]

2. I anticipate physical health problems will limit my physical activities [Insert time frame: 1 week, 1 month, 3 months, 6 months after surgery]:
    a. Not at all
    b. Very little
    c. Somewhat
    d. Quite a lot
    e. Will not be able to do physical activities [Domain: Physical functioning]

3. How much difficulty do you anticipate having doing your daily work, both at home and away from home, because of your physical health [Insert time frame: 1 week, 1 month, 3 months, 6 months after surgery]?
    a. None
    b. Very little
    c. Some
    d. Quite a lot
    e. Will not be able to do daily work [Domain: Physical role functioning]

4. How much bodily pain do you anticipate having [Insert time frame: 1 week, 1 month, 3 months, 6 months after surgery]?
    a. None
    b. Very mild
    c. Mild
    d. Moderate
    e. Severe
    f. Very severe [Domain: Pain]

5. How much energy do you anticipate having [Insert time frame: 1 week, 1 month, 3 months, 6 months after surgery]?
    a. Very much
    b. Quite a lot
    c. Some
    d. A little
    e. None [Domain: Vitality]

6. How much do you anticipate your physical health or emotional problems will limit your usual social activities with family or friends [Insert time frame: 1 week, 1 month, 3 months, 6 months after surgery]?
    a. Not at all
    b. Very little
    c. Somewhat

    d. Quite a lot
    e. Will not be able to do social activities [Domain: Social role functioning]

7. [Insert time frame: 1 week, 1 month, 3 months, 6 months, 1 year] after starting treatment, how much do you anticipate that you will be bothered by emotional problems (such as feeling anxious, depressed, or irritable)?
    a. Not at all
    b. Slightly
    c. Moderately
    d. Quite a lot
    e. Extremely [Domain: Mental health]

8. How much do you anticipate personal or emotional problems will keep you from doing your usual work, school or other daily activities [Insert time frame: 1 week, 1 month, 3 months, 6 months after surgery]?
    a. Not at all
    b. Very little
    c. Somewhat
    d. Quite a lot
    e. Could not do daily activities [Domain: Emotional role functioning]
